# Cross‐tissue immune profiling of *APOE* ε4 reveals early dysregulation in Alzheimer's disease

**DOI:** 10.1002/alz.71714

**Published:** 2026-08-31

**Authors:** Artur Shvetcov, Shannon Thomson, Mark E. Graham, Brittany Hauger, Jessica E. Keller, Christine J. Smoyer, Sarah E. Tague, Ann‐Na Cho, Farhad B. Imam, Varsha Krish, Global Neurodegeneration Proteomics Consortium (GNPC), Matthew K. Taylor, Jonathan D. Mahnken, Debra K. Sullivan, Joanne H. Reed, Jeffrey M. Burns, Chad Slawson, Russell H. Swerdlow, Heather M. Wilkins, Caitlin A. Finney

**Affiliations:** ^1^ Neurodegeneration and Disease Modelling Laboratory Westmead Institute for Medical Research, The University of Sydney Westmead New South Wales Australia; ^2^ School of Medical Sciences, Faculty of Medicine and Health The University of Sydney Sydney New South Wales Australia; ^3^ Biomedical Proteomics Children's Medical Research Institute, The University of Sydney Westmead New South Wales Australia; ^4^ University of Kansas Alzheimer's Disease Research Centre University of Kansas Medical Center Kansas City Kansas USA; ^5^ Integrative Imaging Core University of Kansas Medical Center Kansas City Kansas USA; ^6^ Human Brain Microphysiology Systems Group, School of Biomedical Engineering, Faculty of Engineering The University of Sydney Sydney New South Wales Australia; ^7^ Gates Ventures Seattle Washington USA; ^8^ Autoimmunity and Amyloidosis Research Group Westmead Institute for Medical Research, The University of Sydney Westmead New South Wales Australia; ^9^ Department of Neurology University of Kansas School of Medicine Kansas City Kansas USA; ^10^ University of Kansas Cancer Center University of Kansas Medical Center Kansas City Kansas USA; ^11^ Department of Biochemistry and Molecular Biology University of Kansas Medical Center Kansas City Kansas USA; ^12^ Department of Cell Biology and Physiology University of Kansas Medical Center Kansas City Kansas USA

**Keywords:** apolipoprotein E ε4, (*APOE* ε4), cerebrospinal fluid, cortical organoid, dorsolateral prefrontal cortex, inflammation, machine learning, plasma, proteomics, superior temporal gyrus

## Abstract

**INTRODUCTION:**

*Apolipoprotein E (APOE)* ε4 is the strongest genetic risk factor for late‐onset Alzheimer's disease (AD), but its contribution to disease pathogenesis remains incompletely understood.

**METHODS:**

Here, we integrate proteomic profiling of plasma (*n* = 9028), cerebrospinal fluid (*n* = 1099), dorsolateral prefrontal cortex (*n* = 720), and superior temporal gyrus (*n* = 105) to define the immune phenotype associated with *APOE* ε4.

**RESULTS:**

We identify a conserved, allele dose‐dependent pro‐inflammatory immune protein signature across peripheral and central tissues independent of AD diagnosis. This signature also emerges in patient‐derived cortical organoids prior to amyloid beta and tau pathology, supporting a genotype‐driven mechanism. Cross‐tissue comparisons reveal shared innate and antiviral responses alongside tissue‐specific immune signaling. Notably, a 12‐week medical ketogenic diet partially reversed the *APOE* ε4 immune signature.

**DISCUSSION:**

These findings position immune dysregulation as an early and tractable driver of AD risk in *APOE* ε4 carriers with direct implications for targeted prevention strategies.

## Highlights

- Cross‐tissue proteomics maps *APOE* ε4 immune signatures in AD cohorts.
- *APOE* ε4 shows allele dose‐dependent pro‐inflammatory immune activation.
- *APOE* ε4 immune features are recapitulated in cortical organoids.
- Immune signature emerges before Aβ and tau biomarker positivity.
- A ketogenic diet partially remodels the *APOE* ε4‐linked immune signature.

## 1 BACKGROUND

The functional consequences of the largest genetic risk factor for late‐onset Alzheimer's disease (AD), the ε4 variant of the apolipoprotein E (*APOE*) gene, and how they drive AD pathogenesis remain poorly understood. Recent accumulating evidence suggests that *APOE* ε4 exerts broad modulatory effects on both innate and adaptive immunity across peripheral tissues and the central nervous system. *APOE* ε4 is associated with a shift toward a heightened pro‐inflammatory state.^1^, ^2^ This is further evidenced by peripherally derived immune cells showing exaggerated responses to innate immune stimulation,^3^, ^4^ altered antigen presentation,^5^ disrupted T‐cell homeostasis^6^, and signs of accelerated immune aging.^7^ In the brain, *APOE* ε4 carriage is linked to pre‐activated microglial phenotypes,^8^ impaired lipid and autophagic homeostasis,^9^, ^10^ and amplified responses to amyloid beta (Aβ) pathology.^10^, ^11^ Together, these findings highlight a genotype‐dependent dysregulation of immune homeostasis in *APOE* ε4 carriers. However, several key questions remain, including whether immune changes represent innate genetic effects or secondary responses to disease, how these signatures evolve temporally with progression to AD, which aspects are systemic, central, or peripheral in origin, and whether they are modifiable. Importantly, while large‐scale clinical proteomics can yield hundreds to thousands of candidate biomarkers and putative therapeutic targets, their translational utility ultimately depends on whether these signatures can be faithfully recapitulated in tractable experimental systems. In the absence of in vitro models that reliably mirror salient features of the human phenotype, mechanistic validation and preclinical testing remain speculative. A critical and often overlooked question, therefore, is whether patient‐derived models can robustly reproduce in vivo molecular phenotypes and serve as effective platforms for biomarker verification and therapeutic interrogation. Resolving these questions is essential to understanding *APOE* ε4‐driven mechanisms of neurodegeneration and developing precision biomarkers and early intervention strategies for AD.

To address how *APOE* ε4 influences immune function across tissues and time, we systematically profiled the plasma, CSF, and brain proteomes of APOE ε4 carriers and non‐carriers with and without AD. We identified a conserved, allele dose‐dependent pro‐inflammatory immune signature spanning both the periphery and central nervous system (CNS), independent of disease. This signature was recapitulated in patient stem cell‐derived cortical organoids and emerged prior to the development of Aβ and tau pathology. This highlights that patient‐derived in vitro models can recapitulate in vivo molecular phenotypes. It also indicates that *APOE* ε4‐driven immune changes are early and intrinsic and may initiate downstream neurodegenerative processes. Cross‐tissue comparisons revealed that many *APOE* ε4‐associated pathways were systemic, reflecting broad innate and adaptive immune activation. Others were tissue‐specific and localized to the CNS or periphery. Importantly, short‐term treatment with an anti‐inflammatory medical ketogenic diet in *APOE* ε4 carriers with AD partially reprogrammed this immune phenotype, reducing pro‐inflammatory signaling and promoting regulatory and tissue‐supportive immune functions. Together, these findings demonstrate that *APOE* ε4 confers a systemic but locally modulated pro‐inflammatory immune phenotype that emerges early, evolves with disease progression, and may promote AD pathology. This provides new insight into *APOE* ε4‐driven mechanisms of neurodegeneration and highlights the potential of this immune state to be therapeutically modulated.

## 2 METHODS

### 2.1 Participants

#### 2.1.1 Global Neurodegeneration Proteomics Consortium (GNPC) cohort

The GNPC cohort is the largest collection of proteomic data for individuals with neurodegenerative diseases and non‐impaired controls from over 20 clinical sites from across the United States, United Kingdom (UK), and Europe. In this study, we included non‐impaired controls (*n* = 6672) and individuals with AD (*n* = 3455). Across both groups, *n* = 6107 were non‐*APOE* ε4 carriers and *n* = 4767 had at least one *APOE* ε4 allele (Table S1). AD was clinically diagnosed within each study site as previously described including a Clinical Dementia Rating (CDR) score of ≥ 1, Mini‐Mental State Examination (MMSE) ≤ 24, and/or Montreal Cognitive Assessment (MoCA) score of ≤23.^12^ Individual plasma and CSF samples, demographic, and clinical variables were collected at a single time point (Table S1). Participants from each study site in the GNPC cohort provided written informed consent, and studies were approved by the relevant institution's ethics committee.^12^

**RESEARCH IN CONTEXT**

1. **Systematic review**: We searched the relevant literature using traditional sources like PubMed for studies examining *APOE* ε4‐related proteomic changes across plasma, CSF, brain tissue, and patient‐derived cellular models. Existing work indicates immune activation and complement dysregulation in *APOE* ε4 carriers but provides limited cross‐tissue integration and few data on modifiability of these phenotypes.
2. **Interpretation**: By integrating plasma, CSF, and brain proteomics with *APOE* genotyped cortical organoids and a ketogenic diet intervention, we identify a conserved, allele dose‐dependent pro‐inflammatory signature in *APOE* ε4 carriers. This signature arises before marked Aβ and tau pathology, shows both systemic and CNS‐specific components, and demonstrates partial remodeling with dietary intervention, suggesting that *APOE* ε4‐associated immune profiles may be both early and responsive to intervention.
3. **Future directions**: Priorities include longitudinal validation of this immune signature for risk stratification, experimental dissection of mechanisms, and targeted immunomodulatory trials in *APOE* ε4 carriers.

#### 2.1.2 Accelerating Medicines in AD Partnership (AMP‐AD) Diverse Cohorts study

The AMP‐AD Diverse Cohorts study is a cross‐consortium project that created harmonized high‐throughput multi‐omics data for *post mortem* brain tissue samples. AD cases were pathologically defined as described elsewhere.^13^ Samples from the dorsolateral prefrontal cortex (dlPFC) and superior temporal gyrus (STG) were collected across four institutions: Mayo Clinic, Rush University, Mount Sinai University Hospital, and Emory University. In this study, we included dlPFC data from *n* = 190 non‐impaired controls and *n* = 530 AD cases. For the STG, *n* = 49 non‐impaired controls, and *n* = 161 AD cases were included. Table S2 lists the demographic characteristics of the included donors. Participants from each study site in the AMP‐AD Diverse Cohorts study provided written informed consent, and studies were approved by the relevant institution's ethics committee.

#### 2.1.3 University of Kansas Alzheimer's Disease Research Center donors for cortical organoids

Two donors from the University of Kansas Alzheimer's Disease Research Center provided *post mortem* skin samples. The non‐carrier donor was a 72‐year‐old female with an *APOE* ε3/ε2 genotype, and the *APOE* ε4 carrier donor was an 84‐year‐old female with an *APOE* ε3/ε4 genotype. Donors were members of the University of Kansas Alzheimer's Disease Center Clinical Cohort, who consented to donation upon their death. This study was reviewed and approved by the University of Kansas Medical Center's Institutional Review Board (IRB) (FWA00003411). All participants provided written informed consent prior to enrollment in the study.

#### 2.1.4 Therapeutic Diets in Alzheimer's Disease (TDAD) clinical trial

The TDAD clinical trial is a single‐blind, randomized, controlled study of the effects of a 12‐week medical ketogenic dietary intervention in patients with mild AD. Participants were recruited through the Clinical Translational Science Unit at the University of Kansas Medical Center Clinical Research Center. Fifteen participants (*n* = 9 *APOE* ε4 carriers and *n* = 6 non‐carriers) were given a ketogenic diet intervention. All participants met the criteria for probable AD based on the National Institute on Aging‐Alzheimer's Association (NIA‐AA) criteria^14^ and had a baseline CDR of 0.5 to 1 (see Table S3 for demographics). Participants with moderate to severe AD, including those residing in a nursing home or dementia special care unit, were excluded from the trial based on our previous experience showing that these individuals had a low retention rate to dietary interventions.^15^ Participants with serious medical conditions, those participating in another clinical trial, women of child‐bearing capacity who were seeking to become pregnant, and non‐English speakers were also excluded. Table S4 lists the inclusion and exclusion criteria for the TDAD clinical trial.

Ketogenic diets focus on increasing quality fat intake and reducing carbohydrate consumption to promote the production of ketone bodies, mimicking the effects of prolonged fasting and vigorous exercise.^16^ Macronutrients were maintained at approximately 70% fat, 20% protein, and ≤10% carbohydrates. A 1:1 emulsified medium chain triglyceride (MCT) oil was also provided to participants in the ketogenic diet group to increase fat intake, enhance ketosis, and increase palatability in line with other medical ketogenic diets.^15^ To reduce gastrointestinal side effects from the MCT, the supplement dose increased on a weekly basis across a 4‐week period, starting from an intake level corresponding to 10% of total fat energy and gradually increasing to 40%. The Mifflin‐St. Jeor equation was used to calculate target energy goals.^17^ All participants received weekly dietary counselling from the study dietitian. Participants also received dietitian‐created ketogenic diet educational material and a manual. Manuals included sample menus, recipes, and strategies for maintaining their respective diet during travel or holidays.

Participants’ *APOE* genotype, medical history, medication use, comorbidities, smoking, and alcohol intake were recorded at their initial screening visit. Blood samples were taken at two time points, a pre‐intervention baseline and after the intervention (12 weeks), for plasma proteomics and to measure the depth of ketosis using a routine serum β‐hydroxybutyrate assay performed commercially by Quest Diagnostics (Lenexa, KS). Participant demographic characteristics are listed in Table S3.

The TDAD clinical trial was approved by the University of Kansas Center IRB (STUDY00143457). Prior to participating in the study, all participants provided informed consent. The trial was registered on ClinicalTrials.gov under registration number NCT03860792.

### 2.2 Cortical organoids

#### 2.2.1 Induced pluripotent stem cell derivation

Induced pluripotent stem cells (iPSCs) were generated from fibroblast samples from both donors using a CytoTune‐iPSC 2.0 Sendai Reprogramming kit (Thermo Fisher Scientific, A16517) according to the manufacturer's instructions. iPSCs were plated down into six‐well plates pre‐treated with 0.08 mg/mL Matrigel (Corning, 356234) in Dulbecco's Phosphate‐Buffered Saline (DPBS). StemFlex medium (Gibco, A3349401) with 100 U/mL penicillin‐streptomycin added (Gibco, 15140122) was added to each well and changed every 2 days. iPSCs were passaged once a week using ReLeSR (StemCell Technologies, 15140122) as per the manufacturer's protocol. Following each passage, medium was supplemented with 0.01 mM Y‐27632 (StemCell Technologies, 72302) for 24 h.

#### 2.2.2 iPSC‐derived microglia differentiation

iPSCs were differentiated into hematopoietic progenitor cells (HPCs) using a STEMdiff Hematopoietic kit (StemCell Technologies, 5310) as per the manufacturer's instructions. First, iPSCs were passaged into HPC Medium A in 24‐well plates pre‐treated with 0.04 mg/mL Matrigel. After 3 days, they were cultured with HPC Medium B for nine more days. Mature HPCs were then collected and passaged into STEMDiff Microglia Differentiation medium (StemCell Technologies, 100‐0019) as per the manufacturer's instructions. Fresh medium was added every other day for 24 days, and cells were passaged once on day 12. After differentiation, microglia were matured in BrainPhys medium with N2‐A and SM1 supplements (StemCell Technologies, 05793) for 4 days prior to integration into embryoid bodies.

#### 2.2.3 Cortical organoid development

iPSCs were first passaged three times to stabilize the line. Embryoid bodies were generated in ultra‐low attachment dishes (Corning, 3261) using StemFlex medium supplemented with 100 U/mL penicillin‐streptomycin and 0.01 mM y‐27632 ROCK inhibitor. Medium was changed every 2 days. Embryoid body medium was replaced into N2/B27 medium (Table S5) and transferred to a 24‐well plate. Mature microglia were added to each well at a concentration of 0.8 × 10^5^ cells per well. Fresh N2/B27 medium was added every 2 days, and organoids were passaged once every 2 weeks. Cortical organoids were aged for either 4 or 8 weeks.

#### 2.2.4 Immunofluorescent staining

At each time point, cortical organoids were removed from the medium and washed in DPBS three times for 10 min. They were then fixed in 4% paraformaldehyde (Sigma‐Aldrich, 158127) for 20 min, washed in DPBS once for 10 min, and stored in cyroprotectant (30% glycerol [Sigma‐Aldrich, G5516], 30% ethylene glycol [Sigma‐Aldrich, 324558], 40% PBS) at −20°C. Organoids were washed in PBS three times for 10 min and incubated with blocking solution (5% donkey serum [Sigma‐Aldrich, D9663] or 5% bovine serum albumin [BSA], 0.1% Triton X‐100 [Sigma‐Aldrich, #X100], and PBS) overnight at 4°C. Organoids were then incubated with primary antibodies (Table S6) made up in blocking solution for 2 days at 4°C. They were then washed three times with PBS for 10 min followed by an overnight incubation with secondary antibodies (Table S6) made up in blocking solution at 4°C. This process was repeated for each primary antibody. The microtubule‐associated protein 2 (MAP2), glial fibrillary acidic protein (GFAP), and ionized calcium‐binding adapter molecule 1 (IBA1) stained organoids were co‐stained for DAPI using NucBlue Live ReadyProbes (Invitrogen, R37605) as per the manufacturer's instructions and incubated for 3 days at 4°C. The Aβ and phosphorylated tau at threonine 217 (p‐tau217) organoids were co‐stained for DAPI using ProLong Gold AntiFade with DAPI (Invitrogen, P36931).

Prior to imaging, stained cortical organoids were washed three times in PBS for 10 min and incubated with CytoVista 3D Cell Culture clearing reagent (Invitrogen, V11315) overnight at 4°C. To visualize and quantify Aβ and p‐tau217 staining, whole cortical organoids were imaged using a Nikon TI2‐E inverted microscope attached to a Yokagawa CSU‐W1 spinning disk confocal microscope with SoRa super resolution. Images were acquired with a Hamamatsu Fusion BT camera using Nikon NIS‐Elements Advanced Research software. To visualize and quantify MAP2, IBA1, and GFAP staining, whole cortical organoids were imaged using a Marianas 3i Spinning Disk Confocal microscope with Super‐Resolution by Optical Re‐Assignment (SoRa). Representative images were taken using Fiji ImageJ version 1.54p.^18^

#### 2.2.5 ELISA for secreted Aβ42/Aβ40 and p‐tau217

Medium was collected from cortical organoids at 4 and 8 weeks of age, and proteins were extracted using acetone precipitation. Briefly, 1 mL of ice‐cold acetone (cooled to −20°C) was added to 0.5 mL of collected medium in a tube. Tubes were vortexed, incubated for 60 min at −20°C, and then centrifuged for 10 min at 15,000 × g. The supernatant was removed and pellets were washed in 70% ethanol before being resuspended in 8 M urea. Secreted human Aβ40 and Aβ42 were measured with an ELISA as per the manufacturer's instructions (Thermo Fisher Scientific, KHB3481 and KHB3544). Secreted human p‐tau217 was also measured with an ELISA as per the manufacturer's instructions (Cell Signaling Technology, 59672). All samples were diluted 1:5, and concentrations were normalized to protein content measured via Pierce BCA protein assay (Thermo Fisher Scientific, 23225). Graphs were made in GraphPad Prism version 10.5.0.

### 2.3 Proteomics

#### 2.3.1 GNPC cohort SomaScan assay

Proteomics of plasma and CSF samples from the GNPC cohort was done using the SomaScan version 4.1 assay (SomaLogic). The SomaScan assay detects approximately 7000 proteins using aptamer‐based technology called slow off‐rate modified aptamers (SOMAmers). These contain chemically modified nucleotides that bind with high specificity and affinity to target proteins.^19^, ^20^ Raw data are provided by SomaLogic following standardization, normalization, and calibration, including adaptive normalization by maximum likelihood (ANML). Protein measurements are provided in relative fluorescent units (RFU). Prior to their inclusion in the GNPC dataset, aptamers are mapped to Uniprot. Details on the creation and harmonization of the GNPC dataset are described elsewhere.^12^ Data from the GNPC dataset were log2 transformed and standardized prior to analyses in the current study and done separately on training and testing datasets.

#### 2.3.2 AMP‐AD Diverse Cohorts study tandem mass tag (TMT) quantitation

Proteomics on *post mortem* dlPFC and STG homogenates was done using tandem mass tag (TMT) mass spectrometry, as previously described.^13^ Using a conservative approach, we only included proteins with ≤30% missing values across samples and imputed these values using the median. Batch effects were accounted for by fitting a linear regression model, as in the original study.^13^

#### 2.3.3 Cortical organoids label‐free mass spectrometry

At 4 and 8 weeks of age, cortical organoids were snap frozen in liquid nitrogen and stored at −20°C. To prepare samples for mass spectrometry, 0.2% n‐dodecyl‐β‐D‐maltoside (DDM) in 50 mM triethylammonium bicarbonate (TEAB) with 5 mM Tris(2‐carboxyethyl)phosphine (TCEP) was added to each organoid. A pestle suitable for a 0.5 mL tube attached to a drill was used to homogenize the organoids for 10 s. The tubes were incubated at 85°C for 10 min, cooled, then incubated for 30 min at 22°C with 10 mM iodoacetamide. The samples were frozen in dry ice and then lyophilized. To each sample was added 10 µL of 0.1% DDM in 50 mM TEAB with 2.5 mM TCEP, 0.5 ug of LysC (WAKO Fujifilm), and 1 ug of trypsin (Sigma, trypzean). The samples were incubated at 42°C for 2 h, then a further 0.5 ug of trypsin was added and incubated at 33°C for 4 h. Each sample was acidified with 0.2 µL of formic acid and diluted with 30 µL of 0.1% trifluoroacetic acid and then desalted using the STAGEtip method.^21^

Each sample was analyzed by liquid chromatography tandem mass spectrometry (LC‐MS/MS) using the Vanquish Neo system and Astral Orbitrap mass spectrometer (Thermo Fisher Scientific). Samples were loaded onto a Pepmap Neo C18 5 µm particle 5 mm long by 300 µm inside diameter trap column (Thermo Fisher Scientific) at up to 10 µL/min and at a maximum pressure of 800 bar. They were then eluted through a 15‐cm‐long 150‐µm‐inside diameter Pepmap C18 EASY‐spray column with 2 µm 100 Å particles (Thermo Fisher Scientific). The column was heated to 40°C using the EASY‐spray ion source operating a 1.9 kV. The S lens radio frequency level was 40, and capillary temperature was 280°C.

The liquid chromatography used buffer A (solution of 0.1% formic acid) and buffer B (0.1% formic acid and 99.9% acetonitrile). After loading the sample in 3.6% buffer A, the gradient at 1 µL/min was from 7.2% to 25.2% buffer B in 19.7 min, to 31.5% buffer B in 3.7 min, to 49.5% buffer B in 0.4 min, and to 90% buffer B at 3 µL/min in 0.5 min and held for 0.7 min. MS acquisition was for 26 min. The MS scans in the orbitrap analyzer were at a resolution of 240,000 with automatic gain control set to 5,000,000 and a maximum ion time of 3 ms for m/z 380 to 980. The data‐independent acquisition MS/MS scans with a window of 2 m/z in the Astral analyzer had automatic gain control at 50,000 for a maximum ion time of 3 ms. The loop was controlled to 0.6 s. The MS/MS scan range was 150 to 2000 m/z. The normalized collision energy was 25.

The raw LC‐MS/MS data were processed with DIA‐NN version 1.9.2.^22^ The *Homo Sapiens* reference proteome downloaded Feb 24 2025 with 20,644 genes, using canonical sequences only, was used to create an in silico library for peptide‐spectrum matching. N‐terminal methionine excision was allowed. Carbamidomethyl (C) was a fixed modification. Peptide length was 7–30. Initial mass accuracy was 10 ppm and MS1 accuracy was 4 pm. Digestion was set to trypsin/P with a maximum of 1 missed cleavage. Precursor false discovery rate was 1%. Match between runs was disabled. Heuristic protein inference was enabled, as were all other default algorithm settings for DIA‐NN version 1.9.2.

#### 2.3.4 TDAD clinical trial SomaScan assay

Proteomics of plasma samples before and after the dietary intervention were done using the SomaScan version 4.1 assay (SomaLogic) that detects approximately 7000 proteins. Raw data were provided by SomaLogic following standardization, normalization, and calibration, including ANML, and mapped to UniProt. Protein measurements are provided in relative fluorescent units (RFU). Data were log2 transformed and standardized prior to analyses in this study.

### 2.4 Statistical analyses

#### 2.4.1 Feature selection

**FIGURE 1:**
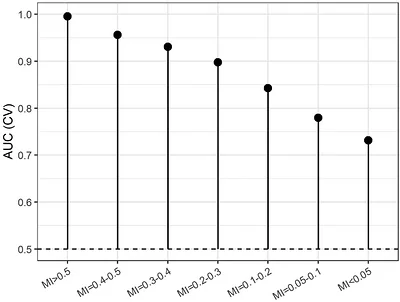
Sensitivity analysis of mutual information (MI) threshold. The area under the curve sharply increases at the interval MI = 0.1 to 0.2, identifying this as an empirical signal/noise breakpoint and a conservative threshold for machine learning analyses.

In the plasma, CSF, dlPFC, and STG, *APOE*4 proteins were identified using mutual information^23^ as previously reported.^1^, ^2^ In plasma and CSF, we identified *APOE* ε4‐specific proteins using all *APOE* ε4 carriers and non‐carriers (both AD and NI controls) to validate our previous findings where *APOE* ε4 proteins were identified only in NI controls.^2^ In the dlPFC and STG, we identified *APOE* ε4 proteins in non‐impaired controls. We performed a mutual information‐based feature prioritization and evaluated performance across mutual information values. Features were binned into six mutual information ranges (<0.05, 0.05 to 0.1, 0.1 to 0.2, 0.3 to 0.4, 0.4 to 0.5, and >0.5) and used as predictors in repeated 5 × 3 cross‐validated decision tree models. The area under the curve (AUC) increased sharply at the interval 0.1 to 0.2 (from 0.79 to 0.86; Figure 1), identifying this value as an empirical signal/noise breakpoint (elbow). Therefore, the mutual information value of ≥0.1 was selected as a conservative threshold for feature inclusion in machine learning analyses. We confirmed our feature selection method using principal component analysis (PCA). For plasma and CSF, we also performed a second feature selection using mutual information on *APOE* ε4 carriers with AD relative to non‐impaired control *APOE* ε4 carriers. Mutual information was calculated in R (version 4.4.1) using the FSelectorRcpp package,^24^ and PCA plots were made using the ggplot2 package.^25^

#### 2.4.2 Machine learning

We used classification and regression trees (CART) and random forest to test the predictive performance of our identified *APOE* ε4 proteins for differentiating between *APOE* ε4 carriers and non‐carriers. The dataset was split into a 70% training and validation set and a 30% withheld (unseen) testing set. Model training and evaluation were done using a five‐fold cross‐validation procedure repeated 10 times. Machine learning was done in R (version 4.4.1) using the caret package.

#### 2.4.3 Cortical organoid proteomics

Label‐free mass spectrometry proteomic data were analyzed in R (version 4.4.1) using the limma package. The raw data consisted of protein group intensities across biological samples, with sample names encoding both experimental condition (*APOE* ε4 carrier or non‐carrier) and time point (4 or 8 weeks). The protein intensity matrix was filtered to retain proteins with valid quantification in at least 30% of samples. Missing values were imputed per protein by replacing missing entries with the minimum observed intensity for that protein, assuming missingness was due to low abundance below the detection limit. Protein intensities were log2 transformed to stabilize variance. Differential protein abundance between *APOE* ε4 and non‐carrier cortical organoid samples was assessed at both 4‐ and 8‐week time points using a linear modeling framework with empirical Bayes moderation, implemented in limma. Specific contrasts (*APOE* ε4 vs non‐carrier at each time point) were tested. Resulting *p* values were corrected for multiple testing using the Benjamini‐Hochberg method to control the false discovery rate (FDR). Proteins with adjusted *p* values (FDR) below 0.05 were considered significantly differentially abundant. Volcano plots were constructed using −log_10_‐transformed adjusted *p* values on the *y*‐axis. Plots show significance thresholds that were applied at FDR‐adjusted *p* < 0.05. All plots and downstream analyses were performed in R using the ggplot2 package.

#### 2.4.4 Cortical organoid AD pathology

ELISA data for secreted Aβ42/40 and p‐tau217 were analyzed at each time point using a two‐tailed unpaired *t*‐test. To analyze interactions between genotype and time, a two‐way ANOVA was used. All statistical analyses were performed in GraphPad Prism (version 10.5.0).

#### 2.4.5 TDAD clinical trial proteomics

Log2‐transformed SomaScan assay data were analyzed in R (version 4.4.1) using the limma package. Differential protein abundance between baseline and after the study in *APOE* ε4 carriers and non‐carriers was assessed using a linear modeling framework with empirical Bayes moderation, implemented in limma. Resulting *p* values were corrected for multiple testing using the Benjamini‐Hochberg method to control the FDR. Proteins with adjusted *p* values (FDR) below 0.05 were considered significantly differentially abundant. Volcano plots were constructed using −log_10_‐transformed adjusted *p* values on the *y*‐axis. Plots show significance thresholds that were applied at FDR‐adjusted *p* < 0.05. All plots and downstream analyses were performed in R using the ggplot2 package.

#### 2.4.6 Enrichment analysis

Enriched biological functions and pathways across *APOE* ε4 proteins were assessed using NetworkAnalyst (version 3.0).^26^, ^27^, ^28^ Protein–protein interactions were identified using a first‐order network in the International Molecular Exchange Consortium (IMEx) interactome database^29^ and InnateDB.^30^ Network enrichments for biological processes and pathways were done using the PANTHER classification system^31^ and Kyoto Encyclopedia of Genes and Genomes (KEGG) database,^32^ respectively. The statistical significance of the enriched networks was determined by FDR > 0.05. Proteins enriched in the KEGG AD pathway were visualized using KEGG mapper.^33^ Cell‐type‐specific descriptive comparative analyses for immune cells, brain regions, and white matter cells were done using single‐cell RNA sequencing data from the Human Protein Atlas (version 23, Ensembl version 109).^34^, ^35^, ^36^ Here, the corresponding protein‐coding transcripts per million for each *APOE* ε4 protein was identified. Expression for each cell type was normalized using min‐max scaling. Heatmaps were generated to visually represent these enrichments and comparisons using R (version 4.4.1).

### 2.5 Data availability

The harmonized GNPC data used to generate these findings were provided to consortium members in June 2024 and will be made available to the public upon request by the AD Data Initiative by July 1, 2025. Members of the global research community will be able to access the metadata and submit a data use request via the AD Discovery Portal (https://discover.alzheimersdata.org/). Access is contingent on adherence to the GNPC Data Use Agreement and the Publication Policies.

The AMP‐AD Diverse Cohorts study data are available through the AD Knowledge Portal (https://adknowledgeportal.synapse.org/). Researchers who wish to access this controlled dataset are required to submit a Data Use Agreement. More information can be found here: https://adknowledgeportal.synapse.org/Data%20Access.

### 2.6 Code availability

All code used in this study is publicly available at https://github.com/Art83/AD_apoe.

## 3 RESULTS

### 3.1 *APOE* ε4 carriers have a distinct pro‐inflammatory immune phenotype in the plasma and CSF

**FIGURE 2:**
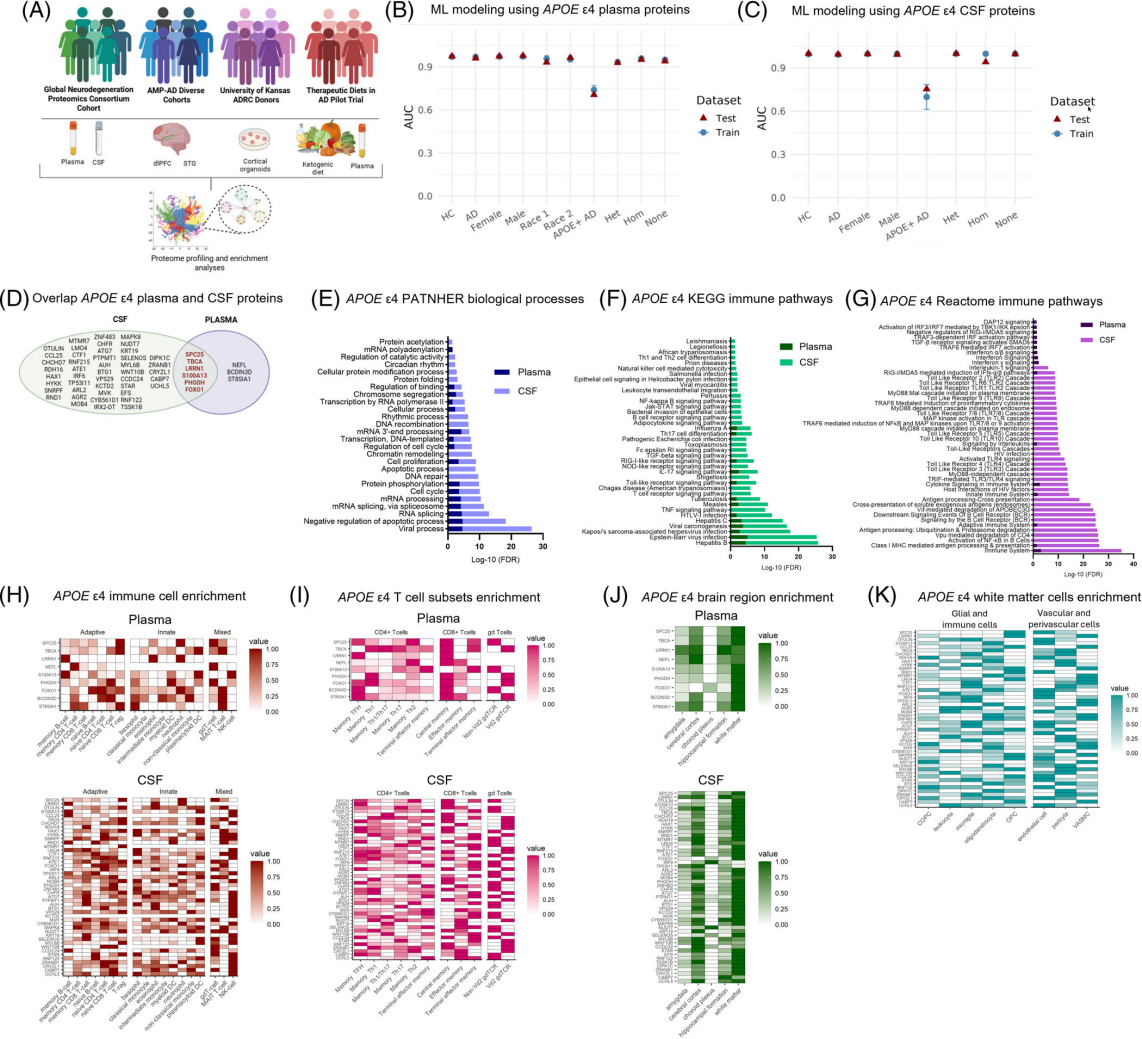
Study design and characterization of the plasma and cerebrospinal fluid (CSF) proteome of *APOE* ε4 carriers and non‐carriers with and without AD. (a) Design of the current study using plasma and CSF proteomic data from the Global Neurodegeneration Proteomics Consortium cohort, brain proteomic data from the dorsolateral prefrontal cortex (dlPFC) and superior temporal gyrus (STG) of donors from the Accelerating Medicines Partnership in Alzheimer's Disease Diverse Cohorts study, cortical organoids generated from donors from the University of Kansas Alzheimer's Disease Research Center cohort, and plasma proteomic data from the Therapeutic Diets in AD pilot clinical trial of 12‐week treatment with an anti‐inflammatory medical ketogenic diet. (b) Performance metrics of machine learning models using *APOE* ε4 plasma proteins as features. The graph shows mean area under the curve (AUC) ± SD for both testing and training datasets across five folds repeated 10 times. Models were trained and validated on a 70% training dataset and tested using a 30% withheld testing dataset. Race 1 represents Black or African American participants, and Race 2 represents American Indian or Alaskan Native participants. (c) Performance metrics of machine learning models using the 51 *APOE* ε4 CSF proteins as features. The graph shows mean AUC ± SD for both testing and training datasets across five folds repeated 10 times. Models were trained and validated on a 70% training dataset and tested using a 30% withheld testing dataset. (d) Venn diagram showing overlap between identified CSF and plasma *APOE* ε4 proteins. (e) Significantly (false discovery rate [FDR] < 0.05) enriched PANTHER biological processes *APOE* ε4 plasma and CSF proteins, with viral processes as the top enriched pathway across both. (f) Significantly (FDR < 0.05) enriched Kyoto Encyclopedia of Genes and Genomes (KEGG) immune pathways for *APOE* ε4 plasma and CSF proteins. Enriched pathways included those involved in pro‐inflammatory, cytokine, infection, and both adaptive and innate immune function. (g) Significantly (FDR < 0.05) enriched Reactome immune pathways for *APOE* ε4 plasma and CSF proteins. Enriched pathways mirror those found with KEGG, with enrichment for pro‐inflammatory, cytokine, and broad adaptive and innate immune functions. (h) Comparison of *APOE* ε4 plasma and CSF proteins across distinct immune cell subsets. Plasma proteins were found across neutrophils, basophils, and T cells. CSF proteins were more enriched in T cells and NK cells. (i) Given that we found many proteins were expressed in T cells, broadly, we did a further comparison for distinct T‐cell subsets. *APOE* ε4 plasma proteins were most found in central memory CD8^+^ T cells and CD4^+^ memory TFH cells. CSF *APOE* ε4 proteins were also found in CD4^+^ memory TFH cells as well as CD4^+^ memory Th1, CD8^+^ terminal effector memory, and non‐Vδ2 γδ T cells. (j) Enrichment of *APOE* ε4 plasma and CSF proteins across commonly affected brain regions in AD, with the highest comparative levels for white matter. (k) Further comparative analysis of *APOE* ε4 CSF proteins across white matter cell subtypes. Microglia and oligodendrocytes were the most involved glial and immune cells and endothelial and vascular cells were the most involved vascular and perivascular cells. Descriptive comparative analyses are based on single‐cell RNA‐sequencing data from the Human Protein Atlas,^34^, ^35^, ^36^ and plots show mix‐max scaling of protein‐coding transcripts per million for each identified *APOE* ε4 plasma protein. AD, Alzheimer's disease; APOE, apolipoprotein E; COPC, committed oligodendrocyte precursor cell; CSF, cerebrospinal fluid; DC, dendritic cell; dlPFC, dorsolateral prefrontal cortex; Het, heterozygous; Hom, homozygous; NI, non‐impaired control; OPC, oligodendrocyte precursor cell; STG, superior temporal gyrus; TFH, T follicular helper; VASMC, vascular‐associated smooth muscle cells.

We profiled 6340 plasma proteins of *n* = 2929 AD and *n* = 6099 non‐impaired controls from the GNPC using the SomaScan 7k assay (Table S1 and Figure 2a). Nine *APOE* ε4‐associated plasma proteins were identified (mutual information > 0.1), which clustered by carrier status and allele dose (Table S7 and Figure S1a–c). Machine learning using CART showed these proteins robustly classified *APOE* ε4 carriers versus non‐carriers (AUC > 0.91 across subgroups; Table S8 and Figure 2b), independently of AD status, sex, or race. No plasma proteins differentiated AD *APOE* ε4 carriers from non‐impaired carriers (mutual information < 0.01; AUC = 0.70), confirming an AD‐independent signature. Multi‐class CART further distinguished heterozygous from homozygous carriers (AUC > 0.93).

We then analyzed 6340 CSF proteins from *n* = 526 AD and *n* = 573 non‐impaired controls (Table S1 and Figure 2a), identifying 51 *APOE* ε4‐associated proteins (mutual information > 0.1) that similarly clustered by carrier status and allele dose (Table S9 and Figure S2d–f). CART models accurately classified *APOE* ε4 carriers (AUC = 0.99), again independently of AD status or sex, and distinguished between heterozygous and homozygous individuals (AUC > 0.94; Table S10 and Figure 2c). As in plasma, no CSF proteins distinguished AD from non‐impaired *APOE* ε4 carriers (mutual information < 0.01; AUC = 0.75), further supporting an AD‐independent immune signature.

Six of the nine plasma proteins overlapped with CSF *APOE* ε4 proteins (Figure 2d). Pathway analyses revealed significant enrichment for viral processes (FDR < 0.05) in both plasma and CSF (PANTHER; Figure 2e and Table S11). KEGG and Reactome analyses confirmed enrichment for immune pathways including interferon, interleukin (IL), Th17, transforming growth factor‐β (TGF‐β) signaling, Epstein‐Barr virus, and hepatitis (Figure 2e–f and Table S11). CSF‐specific pathways included toll‐like receptor signaling and NK/B cell pathways, while plasma‐specific enrichments were dominated by interferon signaling (Figure 2e–f and Table S11). Pathway enrichment analysis stratified by direction of differential expression revealed that the majority of significantly enriched immune‐related pathways, across both KEGG and Reactome, were driven by upregulated proteins. Several pathways, however, showed overlapping enrichment among both up‐ and downregulated protein sets (Figure S2A,B). Cell‐type enrichment using Human Protein Atlas single‐cell RNA‐sequencing data^34^, ^35^ showed plasma *APOE* ε4 proteins were preferentially found in innate immune cells (neutrophils, basophils) and T cells, particularly central memory CD8^+^ and CD4^+^ T follicular helper (TFH) T‐cell subsets (Figure 2h–i). CSF proteins mapped most strongly to NK cells and adaptive T‐cell subsets, including CD4^+^ Th1 and CD8^+^ terminal effector memory cells (Figure 2i). Both plasma and CSF *APOE* ε4 proteins were preferentially found in white matter, followed by microglia, oligodendrocytes, and vascular‐associated cells in CSF (Figure 2j–k).

Together, these findings define a robust, AD‐independent, allele dose‐sensitive immune signature in *APOE* ε4 carriers that spans the plasma and CNS and is enriched in adaptive immune and white matter‐resident cell types.

### 3.2 The *APOE* ε4 phenotype extends into the brain, with the STG especially affected

**FIGURE 3:**
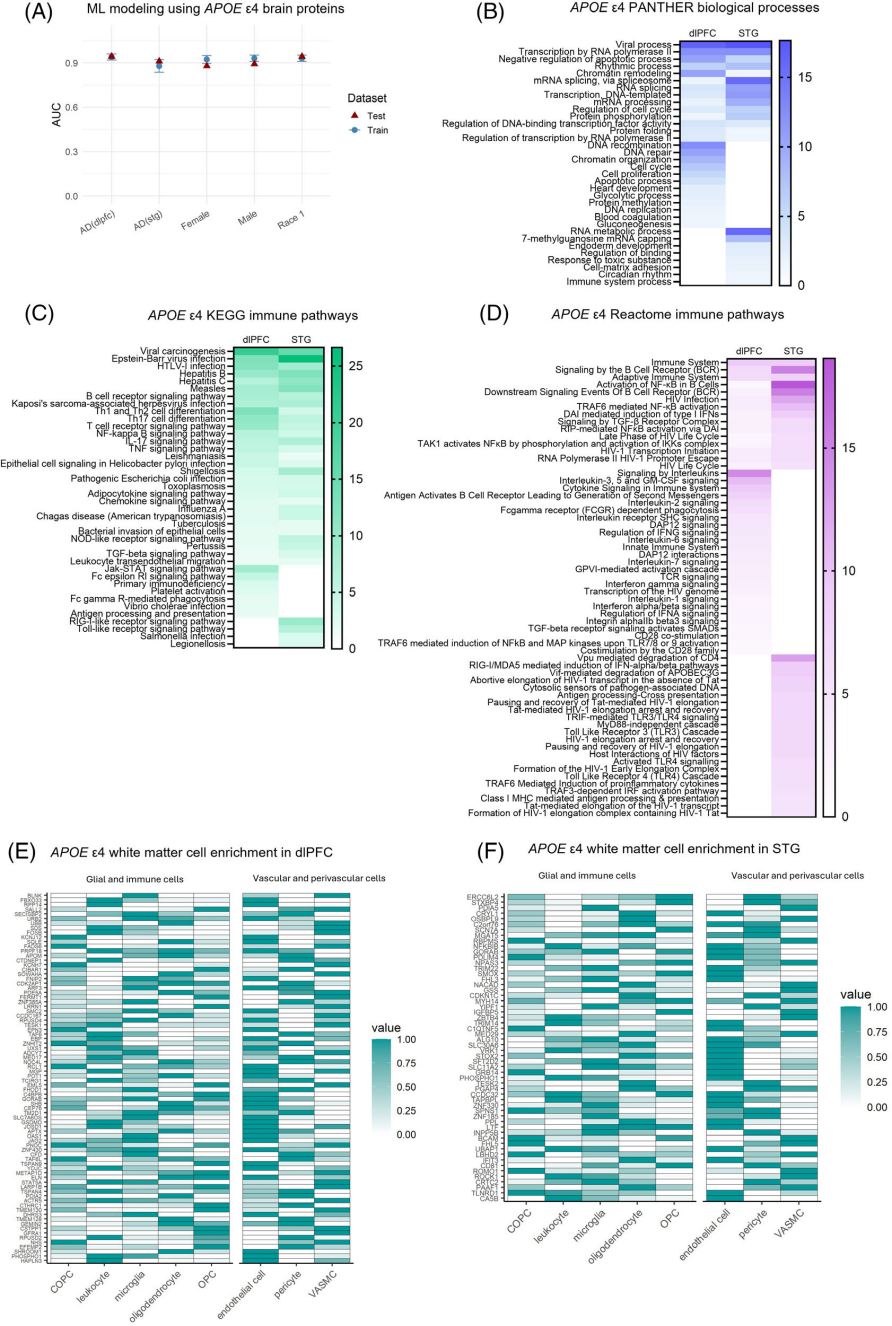
Characterization of dorsolateral prefrontal cortex and superior temporal gyrus brain proteomes of *APOE* ε4 carriers and non‐carriers with and without Alzheimer's disease. (a) Performance metrics of machine learning models using *APOE* ε4 dorsolateral prefrontal cortex (dlPFC) or superior temporal gyrus (STG) proteins as features. The graph shows mean area under the curve (AUC) ± SD for both testing and training datasets across five folds repeated 10 times. Models were trained and validated on a 70% training dataset and tested using a 30% withheld testing dataset. Race 1 refers to Black or African American donors. (b) Significantly (FDR < 0.05) enriched PANTHER biological processes for *APOE* ε4 dlPFC and STG proteins. The top enriched biological processes across both regions were viral processes (FDR = 3.95E‐17 and 2.18E‐18, respectively). The scale bar represents −log10(FDR). (c) Significantly (FDR < 0.05) enriched Kyoto Encyclopedia of Genes and Genomes (KEGG) immune pathways for *APOE* ε4 dlPFC and STG proteins. Enriched pathways included those involved in pro‐inflammatory, cytokine, infection, and both adaptive and innate immune function. The scale bar represents −log10(FDR). (d) Significantly (FDR < 0.05) enriched Reactome immune pathways for *APOE* ε4 dlPFC and STG proteins. Enriched pathways mirror those found with KEGG, with enrichment for pro‐inflammatory, cytokine, and broad adaptive and innate immune functions. Enrichment for these pathways was more significant in the STG than the dlPFC. The scale bar represents −log10(FDR). (e and f) Given our previous findings in the plasma and CSF showing *APOE* ε4 proteins were preferentially found in white matter, we performed a comparative analysis for white matter cell subtypes. (e) dlPFC *APOE* ε4 proteins were found in microglia as well as endothelial cells. (f) STG *APOE* ε4 proteins were similarly found in microglia and endothelial cells. Descriptive comparative analyses are based on single‐cell RNA‐sequencing data from the Human Protein Atlas,^36^ and plots show mix‐max scaling of protein‐coding transcripts per million for each identified *APOE* ε4 dlPFC or STG protein. AD, Alzheimer's disease; APOE, apolipoprotein E; COPC, committed oligodendrocyte precursor cell; CSF, cerebrospinal fluid; dlPFC, dorsolateral prefrontal cortex; NI, non‐impaired control; OPC, oligodendrocyte precursor cell; STG, superior temporal gyrus; VASMC, vascular‐associated smooth muscle cells.

To assess whether the *APOE* ε4‐associated immune phenotype extended into the brain, we analyzed TMT‐based proteomic data from the Accelerating Medicine Partnerships in AD (AMP‐AD) Diverse Cohorts study in the dlPFC (*n* = 530 AD, *n* = 190 non‐impaired controls) and STG (*n* = 161 AD, *n* = 49 non‐impaired controls; Table S2). We identified 85 *APOE* ε4‐associated proteins in dlPFC and 56 in STG of non‐impaired controls (mutual information > 0.1; Table S12), with only two proteins overlapping across regions, GORAB and PHOSPHO1, suggesting region‐specific effects of *APOE* ε4. Coverage differences between SomaScan and TMT precluded direct plasma/CSF comparisons. Random forest modeling showed that *APOE* ε4 brain proteins reliably classified *APOE* ε4 carriers versus non‐carriers with AD (dlPFC AUC = 0.94; STG AUC = 0.91), independently of sex and race for dlPFC (AUC > 0.88; Table S13 and Figure 3a). Insufficient sample size precluded subgroup analysis in STG.

Consistent with plasma and CSF findings, viral processes were the most significantly enriched *APOE* ε4‐associated biological processes (PANTHER; Figure 3b and Table S14). KEGG analyses revealed significant enrichment for pro‐inflammatory, infection‐related, and adaptive immune pathways. There were also region‐specific pathways, including Fc receptor and platelet activation in dlPFC and toll‐like receptor (TLR) and RIG‐I‐like receptor (RLR) signaling in STG (Figure 3c and Table S14). Reactome pathways overlapped across regions for adaptive immune, B‐cell receptor, and human immunodeficient (HIV)‐related pathways. dlPFC‐specific pathways reflected immune cell activation and cytokine regulation, whereas STG pathways were more focused on pathogen sensing and antiviral defense (Figure 3d and Table S14). Stratified pathway enrichment revealed region‐specific directionality in *APOE* ε4‐associated changes. While immune‐related pathways were significantly enriched in both brain regions, the directionality of the contributing proteins diverged. In the dlPFC, immune pathways were driven predominantly by upregulated proteins, whereas in the STG, the same pathways were enriched among downregulated proteins (Figure S3a–d). This suggests region‐specific immune states in *APOE* ε4 carriers, with the dlPFC exhibiting immune activation and the STG potentially reflecting immune suppression, exhaustion, or loss of immune‐competent cell types. Across both regions, *APOE* ε4 brain proteins were preferentially found in microglia and endothelial cells (Figure 3e).

These findings define distinct, region‐specific *APOE* ε4‐associated brain proteomic signatures that recapitulate the viral, pro‐inflammatory, and adaptive immune pathway enrichment previously observed in plasma and CSF. Despite shared immune pathway enrichment, the directionality of protein changes diverged across regions, suggesting differential immune states in the *APOE* ε4 brain. Enrichment of *APOE* ε4‐regulated proteins in microglia and cerebrovascular endothelial cells implicates white matter and neurovascular immune processes as central features of the *APOE* ε4 brain phenotype.

### 3.3 Stem cell‐derived cortical organoids show the *APOE* ε4 phenotype before Alzheimer's pathology onset

Across two clinical cohorts, we demonstrated that *APOE* ε4 carriers exhibited a pro‐inflammatory immune phenotype both peripherally and centrally. However, it is unknown whether the *APOE* ε4 in vivo molecular phenotype can be recapitulated in a human‐specific in vitro model. Understanding this is of the utmost importance for establishing effective platforms for biomarker verification and interrogating therapeutic targets identified in large clinical cohorts. Further, we previously showed that this phenotype was not correlated with hallmark AD pathologies including Aβ, tau, gliosis, or angiopathy.^2^ However, since these clinical samples are derived from symptomatic individuals or asymptomatic individuals who may harbor subclinical AD pathology, it remains unclear whether this *APOE* ε4 immune phenotype precedes or follows the development of AD pathology, a critical question for guiding diagnosis and treatment in *APOE* ε4 carriers.

To address this issue, we generated cortical organoids^37^, ^38^ from iPSC lines of two female donors from the University of Kansas Alzheimer's Disease Research Center cohort: a heterozygous *APOE* ε4 carrier who developed AD and a non‐carrier control. Our objective was to determine whether the naturally occurring *APOE* ε4 molecular phenotype observed in human tissues could be recapitulated in a real‐world, patient‐derived cortical organoid model. This is a critical first step to establish a platform for translational validation of clinical biomarker and therapeutic target candidates. Notably, our extensive multi‐cohort proteomic analyses showed that the *APOE* ε4 immune signature was conserved across sex, with no significant sex‐specific effects or clustering in plasma, CSF, or brain tissue.

**FIGURE 4:**
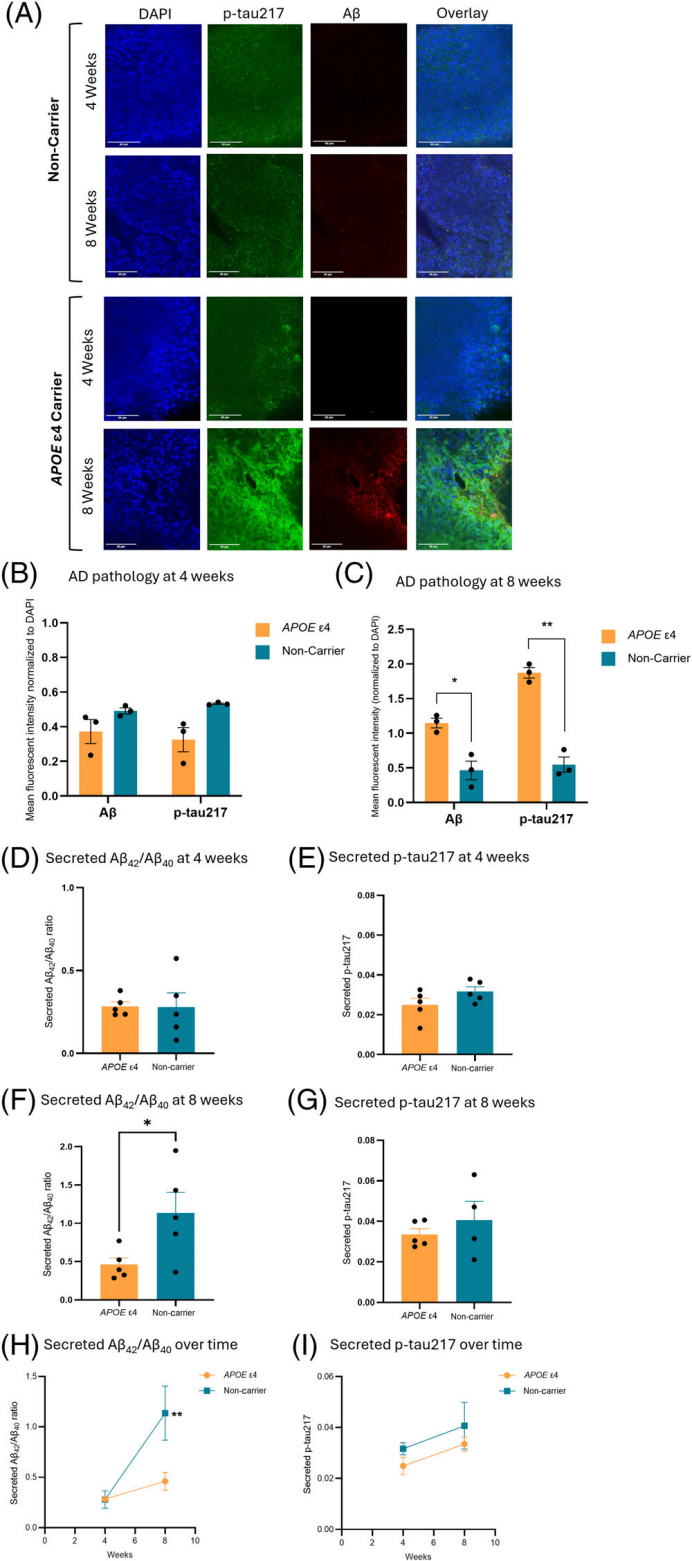
Alzheimer's pathology development in induced pluripotent stem cell‐derived cortical organoids of an *APOE* ε4 carrier and non‐carrier. (a) Representative immunofluorescent confocal microscopy images of the presence of AD pathological markers p‐tau217 and amyloid beta (Aβ) in cortical organoids from an *APOE* ε4 carrier and non‐carrier at 4 and 8 weeks of maturation. (b) Quantification of mean fluorescent intensity of p‐tau217 and Aβ, normalized to DAPI, in cortical organoids from an *APOE* ε4 carrier and non‐carrier at 4 weeks of maturation (*n* = 3 technical replicates/group). (c) Quantification of the mean fluorescent intensity of p‐tau217 and Aβ, normalized to DAPI, in cortical organoids from an *APOE* ε4 carrier and non‐carrier at 4 weeks of maturation (*p = 0.0203, unpaired two‐tailed *t*‐test; **p = 0.0010, unpaired two‐tailed *t*‐test; *n* = 3 technical replicates/group). (d–f) ELISA analysis of secreted Aβ42/40 into the culture medium from *APOE* ε4 carrier and non‐carrier cortical organoids at (d) 4 weeks and (e) 8 weeks of maturation. There was no difference at 4 weeks between the *APOE* ε4 carrier and non‐carrier, whereas the non‐carrier cortical organoids showed significantly (*p = 0.0432, unpaired *t*‐test, *n* = 5 technical replicates/group) higher levels of secreted Aβ42/40 at 8 weeks. (f) The non‐carrier cortical organoids showed significantly different levels of secreted Aβ42/40 over time relative to *APOE* ε4 cortical organoids (**p = 0.0166, two‐way ANOVA, *n* = 5 technical replicates/group). (g–i) ELISA analysis of secreted p‐tau217 into the culture medium from *APOE* ε4 carrier and non‐carrier cortical organoids at (g) 4 weeks and (h) 8 weeks of maturation (*n* = 5 technical replicates/group). There were no significant differences between groups. (i) Time course of *APOE* ε4 carrier and non‐carrier cortical organoid‐secreted p‐tau217 showing no significant changes over time (*n* = 5 technical replicates/group). Error bars on all graphs are representative of SEM.

To better model *APOE* ε4 immune interactions, iPSC‐derived microglia from the same donors were integrated into cortical organoids (Methods; Figure S4). At 4 weeks’ maturation, neither *APOE* ε4 nor non‐carrier organoids showed evidence of p‐tau217 or Aβ accumulation (Figure 4a,b). By 8 weeks, however, *APOE* ε4 organoids exhibited marked accumulation of p‐tau217 (*t*_(2.5)_ = 4.48, *p* = 0.0203, unpaired two‐tailed *t*‐test; Figure 4a,c) and Aβ (*t*_(2.5)_ = 10.02, *p* = 0.0010, unpaired two‐tailed *t*‐test; Figure 4a,c). There were no differences in secreted Aβ42/40 or p‐tau217 at 4 weeks (Figure 4d,e). At 8 weeks, there were significantly reduced levels of secreted Aβ42/40 compared to non‐carriers (*t*_(8)_ = 2.40, *p* = 0.043; unpaired two‐tailed *t*‐test; Figure 4f), consistent with Aβ retention in *APOE* ε4 carrier organoids. Despite intracellular p‐tau217 accumulation in *APOE* ε4 organoids at 8 weeks, there were no significant differences in secreted p‐tau217 (Figure 4g). A significant time × *APOE* genotype interaction in Aβ42/40 secretion was also observed (*F*_(1,8)_ = 9.12, *p* = 0.017; two‐way ANOVA; Figure 4h), and no significant differences were observed in secreted p‐tau217 over time (Figure 4i).

**FIGURE 5:**
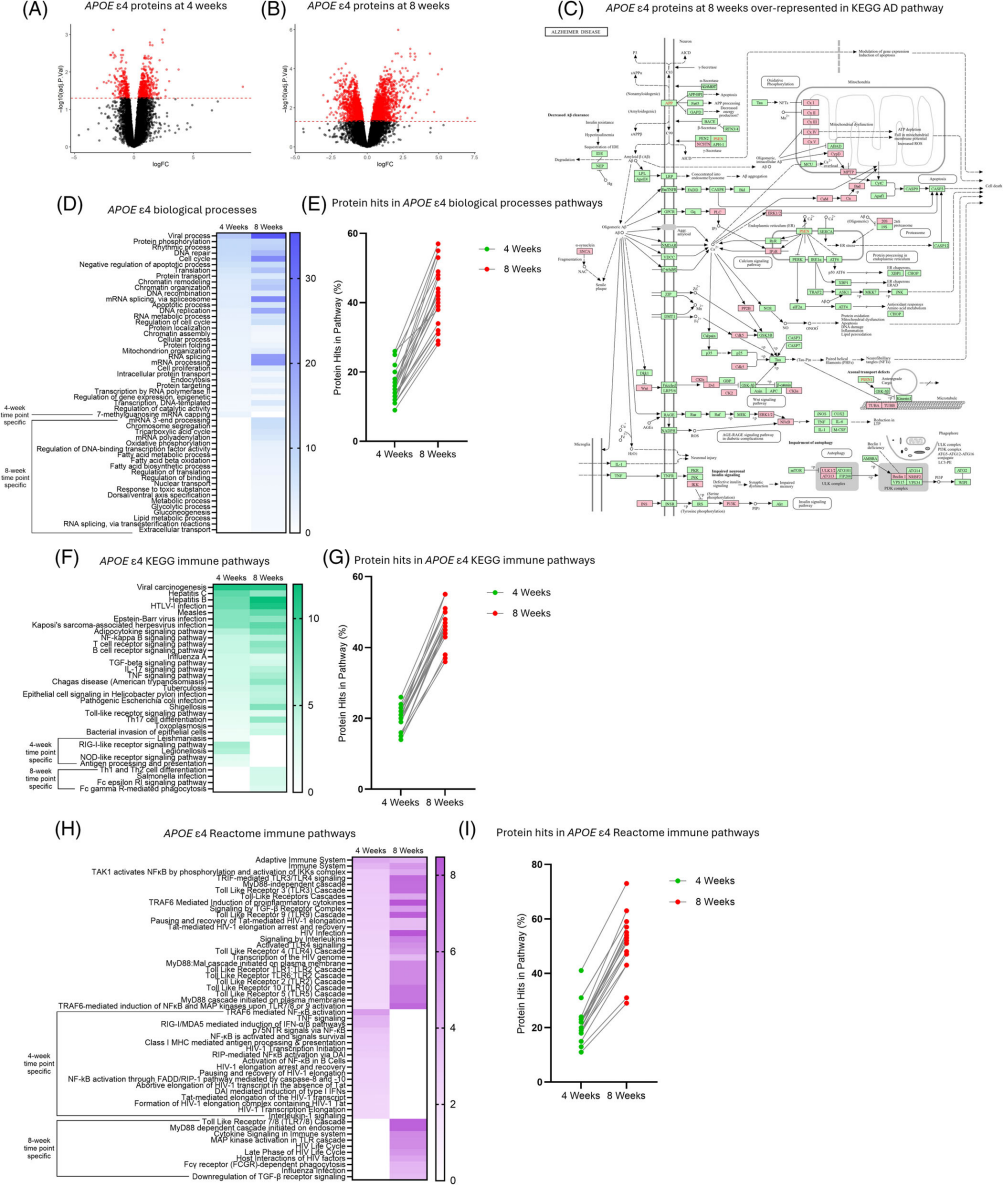
Characterization of *APOE* ε4 proteome over time in stem cell‐derived cortical organoids from an *APOE* ε4 carrier and non‐carrier. (a) Volcano plot representing 642 significantly differentially expressed proteins (in red) in *APOE* ε4 cortical organoids at 4 weeks of maturation before the onset of AD pathologies; *n* = 4 technical replicates/group (see Table S8 for a full list of proteins, adjusted *p* values, and fold changes). (b) Volcano plot representing 2655 significantly differentially expressed proteins (in red) in *APOE* ε4 cortical organoids at 8 weeks of maturation after the onset of AD pathologies; *n* = 4 technical replicates/group (see Table S8 for a full list of proteins, adjusted *p* values, and fold changes). (c) Kyoto Encyclopedia of Genes and Genomes (KEGG) pathway enrichment analysis of differentially expressed proteins in *APOE* ε4 cortical organoids at 8 weeks of maturation showing significant (false discovery rate [FDR] = 0.00000211) enrichment in AD pathway. Pink colored proteins represent those identified as differentially expressed proteins at 8 weeks. (d) Significantly (FDR < 0.05) enriched PANTHER biological processes for *APOE* ε4 cortical organoids at 4 and 8 weeks of maturation. The top enriched process across both time points was viral processes (FDR = 1.29E‐12 and 3.99E‐36, respectively). The scale bar represents −log10(FDR). (e) Graph showing change in number of identified (“hit”) proteins in overlapping *APOE* ε4 PANTHER biological processes across cortical organoids at 4 and 8 weeks of maturation. The *y*‐axis represents the “hit” proteins expressed as a percentage of expected proteins (“hit” proteins in pathway/total number of proteins in pathway). (f) Significantly (FDR < 0.05) enriched KEGG immune pathways for *APOE* ε4 cortical organoids at 4 and 8 weeks of maturation. Enriched pathways were reflective of processes involved in pro‐inflammatory, cytokine, infection, and immune function and timepoint‐specific pathways indicated a changing role of microglia from surveillance and immune sensing to activated and phagocytic. (g) Graph showing change in number of identified (“hit”) proteins in overlapping *APOE* ε4 KEGG immune pathways across cortical organoids at 4 and 8 weeks of maturation. The *y*‐axis represents the “hit” proteins expressed as a percentage of expected proteins (“hit” proteins in pathway/total number of proteins in pathway). (h) Significantly (FDR < 0.05) enriched Reactome immune pathways for *APOE* ε4 cortical organoids at 4 and 8 weeks of maturation. Enriched pathways mirrored those seen in the KEGG analysis and further indicated a change in microglia over time from innate immune sensing and inflammatory priming to phagocytic, disease‐associated microglia (DAM). (i) Graph showing change in number of identified (“hit”) proteins in overlapping *APOE* ε4 Reactome immune pathways across cortical organoids at 4 and 8 weeks of maturation. The *y*‐axis represents the “hit” proteins expressed as a percentage of expected proteins (“hit” proteins in pathway/total number of proteins in pathway).

At 4 weeks of maturation, *APOE* ε4 cortical organoids lacked hallmark AD pathology, providing an opportunity to study early pre‐pathological processes. To investigate how the *APOE* ε4 proteome evolves with disease onset, we performed label‐free mass spectrometry on 10,828 proteins from *APOE* ε4 and non‐carrier organoids at 4 and 8 weeks. At 4 weeks, 642 proteins were differentially expressed between *APOE* ε4 and non‐carriers, increasing to 2655 by 8 weeks (adjusted *p* < 0.05; Table S15 and Figure 5a,b). KEGG enrichment confirmed absence of AD pathway proteins at 4 weeks, with significant enrichment emerging by 8 weeks (FDR = 2.1 × 10^−6^; Figure 5c). Consistent with our plasma, CSF, and brain findings, viral processes were the most enriched biological process at both time points (PANTHER; Figure 5d and Table S16). Pathways evident at 4 weeks were largely exacerbated at 8 weeks, as reflected by increased protein representation (Figure 5e). New 8‐week‐specific enrichments included pathways related to RNA metabolism, oxidative phosphorylation, lipid metabolism, glycolysis, and stress responses, potentially reflecting changes secondary to accumulating pathology (Figure 5d and Table S16).

KEGG immune pathway analysis showed substantial overlap between early and late time points, with progressive intensification over time (Figure 5f,g and Table S16). Early pathways reflected innate immune sensing and antigen presentation, suggesting early *APOE* ε4 microglial priming. By 8 weeks, pathways were reflective of chronic innate activation, phagocytosis, and adaptive immune responses, consistent with a transition to a disease‐associated microglial (DAM) phenotype in response to pathology (Figure 5f and Table S16). Reactome enrichment further supported this finding. Early time points were dominated by NF‐κB, type I interferon, and major histocompatability complex (MHC) class I antigen presentation, while late time points showed increased pathways for phagocytosis, cytokine signaling, and potential astrocyte involvement via TGF‐β signaling (Figure 5h and Table S16). Pathways common to both time points showed progressive activation over time, as evidenced by increased numbers of contributing proteins (Figure 5i). Pathway enrichment analysis stratified by direction of differential expression revealed a pronounced pro‐inflammatory signature in cortical organoids, with most enriched pathways driven by upregulated proteins (Figure S5a–d). In contrast, only a limited number of Reactome pathways were specific to downregulated proteins, primarily related to HIV transcriptional processes, consistent with broader dysregulation of DNA‐ and RNA‐associated biological functions (Figure S5b,d).

Collectively, these data demonstrate that *APOE* ε4 cortical organoids display an early pro‐inflammatory, viral‐related immune phenotype prior to the onset of AD pathology, which progressively intensifies with maturation and disease development. The observed transition from innate immune priming to sustained activation and phagocytic responses is consistent with the emergence of DAM. Stratified enrichment analyses further revealed that this immune activation was predominantly driven by upregulated proteins, whereas downregulated proteins were enriched for HIV‐related transcriptional pathways, implicating the dysregulation of host DNA/RNA processes. These results highlight cortical organoids as a valuable model for dissecting temporal immune mechanisms underlying *APOE* ε4‐driven AD pathogenesis.

### 3.4 Cross‐tissue proteomic analysis reveals systemic as well as central‐ and peripheral‐specific dysregulated pathways in *APOE* ε4 carriers

Thus far, we have independently characterized the proteomes of plasma, CSF, two brain regions, and cortical organoids from *APOE* ε4 carriers and non‐carriers. While broad enrichment of pro‐inflammatory immune pathways was observed across tissues, it remains unclear which *APOE* ε4‐associated molecular changes are systemic versus tissue‐specific. To address this, we performed a cross‐tissue comparison of enriched KEGG and Reactome immune pathways to delineate molecular processes that are systemic, CNS‐specific, or peripheral in nature and to further assess the fidelity of the organoid model in capturing in vivo‐relevant immune signatures.

**FIGURE 6:**
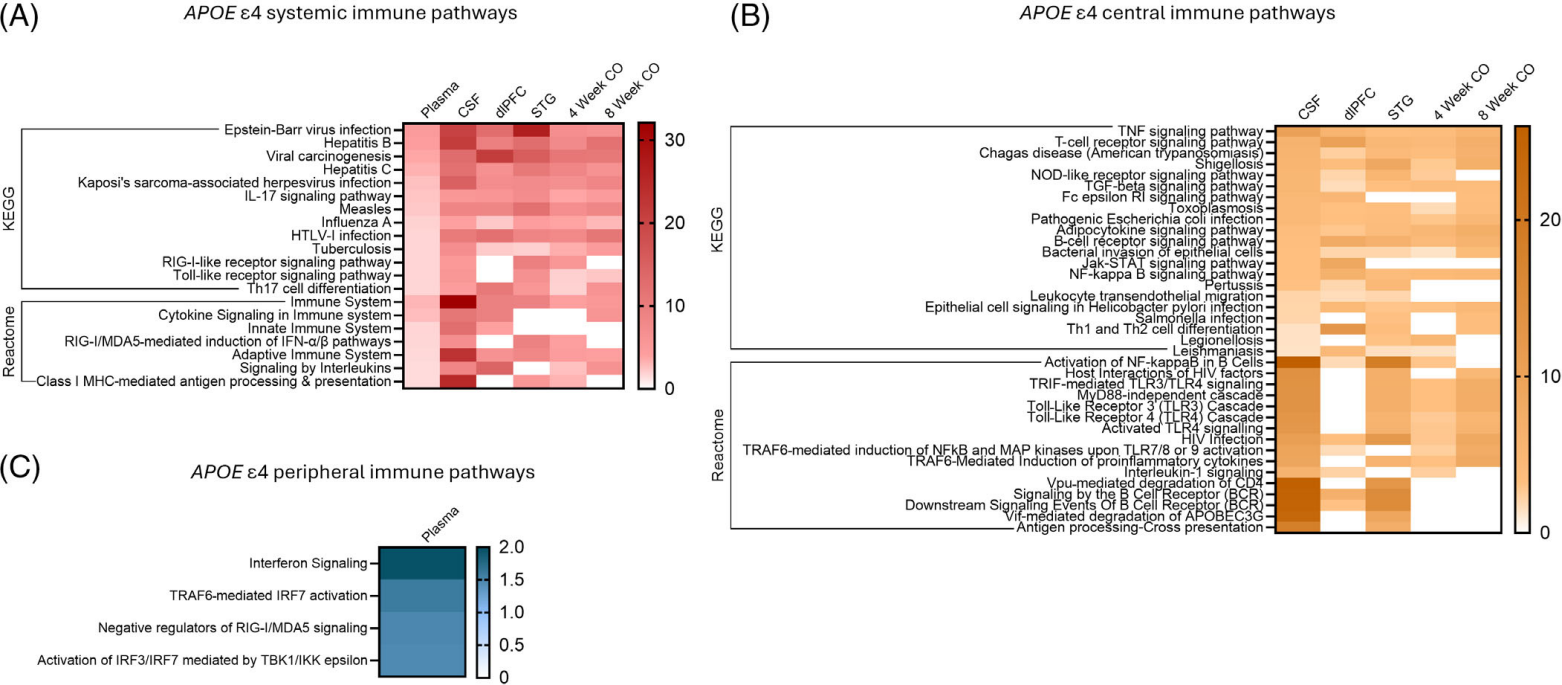
Cross‐tissue comparison of immune pathways in *APOE* ε4 carriers. (a) Systemic *APOE* ε4 immune Kyoto Encyclopedia of Genes and Genomes (KEGG) and Reactome pathways identified across the plasma, cerebrospinal fluid (CSF), dorsolateral prefrontal cortex (dlPFC), superior temporal gyrus (STG), and cortical organoids (CO). (b) Central nervous system‐specific *APOE* ε4 immune KEGG and Reactome pathways identified across the CSF, dlPFC, STG, and CO. (c) Peripheral system‐specific *APOE* ε4 immune KEGG and Reactome pathways identified only in plasma. Scale bars represent −1og10(FDR). AD, Alzheimer's disease; APOE, apolipoprotein E; CO, cortical organoids; CSF, cerebrospinal fluid; dlPFC, dorsolateral prefrontal cortex; FDR, false discovery rate; STG: superior temporal gyrus.

Systemic *APOE* ε4 pathways were broadly representative of conserved innate and adaptive immune responses to diverse viral and bacterial infections and pro‐inflammatory stimuli, as well as generalized cytokine signaling and antigen presentation (Figure 6a). Enrichment was also observed for pattern recognition receptor signaling, interferon responses, and MHC class I antigen presentation, core elements of viral sensing, antiviral immunity, and systemic immune activation (Figure 6a). In contrast, CNS‐specific pathways were associated with more complex immune functions, including adaptive immune signaling through T and B cells, intracellular pathogen responses, chronic inflammatory responses via tumor necrosis factor (TNF), NF‐κB, and JAK‐STAT pathways, and immune modulation (Figure 6b). Enrichment of pathways involved in immune–microbe interactions at tissue barriers and immune cell recruitment was also observed, potentially reflecting infiltrating adaptive immune cells or blood‐brain barrier (BBB)‐associated immune processes (Figure 6b). Notably, five central *APOE* ε4‐enriched pathways were absent in cortical organoids at both time points (Figure 6b), suggesting that these pathways, particularly those requiring mature T/B cell interactions, antigen presentation, and immune cell crosstalk, cannot be fully recapitulated in organoids due to the absence of adaptive, peripherally derived immune cells. Only four peripheral‐only pathways were identified in plasma, primarily involving the fine‐tuning and regulation of interferon‐driven transcriptional responses (Figure 6c).

These results demonstrate that the *APOE* ε4 pro‐inflammatory immune phenotype includes both systemic and CNS‐specific components, with core antiviral and innate immune pathways shared across tissues and more complex adaptive immune and BBB‐associated processes enriched in the CNS. The absence of certain adaptive pathways in organoids further underscores the importance of immune cell interactions in shaping the *APOE* ε4 CNS immune environment. Notably, the organoid model faithfully recapitulated many of the conserved innate and antiviral immune pathways identified in plasma, CSF, and brain, supporting its validity as a tractable human system for studying early *APOE* ε4‐driven immune mechanisms. Together, these findings highlight a multi‐tissue, pro‐inflammatory immune phenotype that may contribute to *APOE* ε4‐driven AD risk.

### 3.5 Short‐term treatment with an anti‐inflammatory ketogenic diet can modulate the pro‐inflammatory phenotype of *APOE* ε4 carriers

*APOE* ε4 carriers have a pro‐inflammatory phenotype that spans both the periphery and CNS. To test whether this phenotype was modifiable, we analyzed plasma samples from a 12‐week pilot clinical trial of a medical ketogenic diet in 15 individuals with mild AD (*n* = 9 *APOE* ε4 carriers, *n* = 6 non‐carriers; Table S3). Medical ketogenic diets, focused on increasingly quality fat intake and reducing carbohydrate consumption to promote production of ketone bodies are widely regarded as a gold‐standard systemic anti‐inflammatory diet.^16^ They have demonstrated treatment benefits for epilepsy, general neurological conditions including AD, and cancers.^15^, ^39^, ^40^, ^41^ The intervention increased serum β‐hydroxybutyrate in both groups, confirming ketone body induction (Table S13).

**FIGURE 7:**
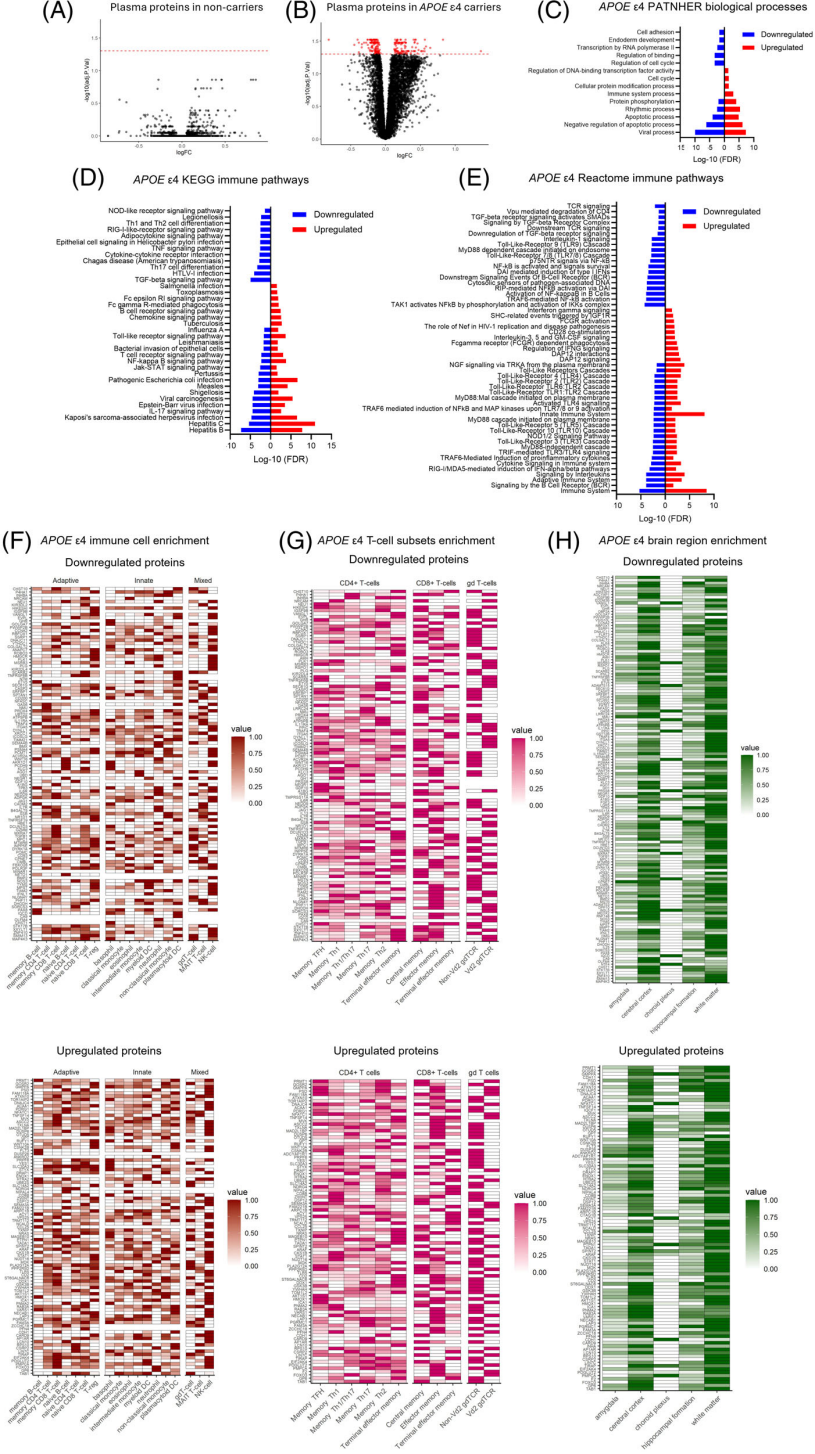
Characterization of plasma proteome of Alzheimer's patients after 12 weeks of an anti‐inflammatory medical ketogenic diet from the Therapeutic Diets in Alzheimer's Disease clinical trial. (a) Volcano plot representing no significantly differentially expressed proteins in non‐carriers at the end of the 12‐week study; *n* = 9 *APOE* ε4 carriers and *n* = 6 non‐carriers. (b) Volcano plot representing 264 significantly (adjusted *p* value < 0.05) differentially expressed proteins in *APOE* ε4 carriers at the end of the 12‐week study; *n* = 9 *APOE* ε4 carriers and *n* = 6 non‐carriers (see Table S11 for a full list of proteins, adjusted *p* values, and fold changes). (c) PANTHER biological processes analysis of pathways enriched for up‐ or downregulated differentially expressed proteins in *APOE* ε4 carriers. (d) KEGG immune pathway analysis of pathways enriched for up‐ or downregulated differentially expressed proteins in *APOE* ε4 carriers. (e) Reactome immune pathway analysis of pathways enriched for up‐ or downregulated differentially expressed proteins in *APOE* ε4 carriers. (f) Descriptive comparative analyses of significantly up‐ or downregulated *APOE* ε4 proteins across distinct immune cell subsets. Downregulated proteins were primarily found in T‐regs and NK cells. Upregulated proteins were primarily enriched across memory CD8^+^ T cells, naïve CD8^+^ T cells, T‐regs, and NK cells. There was also more comparative expression across the spectrum of innate immune cells relative to *APOE* ε4 downregulated proteins. (g) Given that we found high levels in T cells, broadly, we did a further analysis for distinct T‐cell subsets. Downregulated *APOE* ε4 proteins were primarily found in effector memory and central memory CD8^+^ T cells and non‐Vδ2 γδ T cells. Upregulated *APOE* ε4 proteins were similarly found in effector memory CD8^+^ T cells and non‐Vδ2 γδ T cells, as well as CD4^+^ T cells including memory TFH and terminal effector memory cells. (h) Comparative analyses of significantly up‐ and downregulated *APOE* ε4 proteins across commonly affected brain regions in AD, with the highest levels found in white matter. Descriptive comparative analyses are based on single‐cell RNA‐sequencing data from the Human Protein Atlas,^34^, ^35^, ^36^ and plots show mix‐max scaling of protein‐coding transcripts per million for each identified *APOE* ε4 plasma protein. AD, Alzheimer's disease; APOE, apolipoprotein E; DC, dendritic cell; TFH, T follicular helper.

Plasma proteomic profiling (7595 proteins via SomaScan 7k) across baseline and study endpoint revealed no significant changes in non‐carriers (Figure 7a). In contrast, *APOE* ε4 carriers had 264 differentially expressed proteins (161 downregulated, 103 upregulated; adjusted *p* < 0.05; Figure 7b; Table S17). PANTHER analysis identified viral processes as the top enriched biological process across both up‐ and downregulated proteins (Figure 7c and Table S18). A KEGG immune pathway analysis revealed that the medical ketogenic diet led to immune remodeling in *APOE* ε4 carriers rather than broad suppression or activation. Pathways enriched for both up‐ and downregulated proteins included those involved in innate and adaptive pathways (Figure 7d). Pathways enriched in upregulated proteins were associated with adaptive immunity, phagocytosis, and chemotaxis, suggesting enhanced immune surveillance. Downregulated proteins, however, mapped to chronic inflammation and metabolic‐immune signaling, indicating reduced chronic pro‐inflammatory signaling (Figure 7d and Table S18). These findings were mirrored by a Reactome pathway analysis. This showed a bidirectional regulation of TLR and cytokine signaling, with selective upregulation of interferon responses and Fc‐mediated phagocytosis and downregulation of NF‐κB, TGF‐β, TCR, and IL‐1 signaling pathways (Figure 7e and Table S18). Cell‐type comparative analysis showed that both up‐ and downregulated proteins were mapped to T‐regs, NK cells, memory CD8^+^ T cells, and non‐Vδ2 γδ T cells, suggesting functional reprogramming within these populations rather than uniform activation or suppression (Figure 7f,g). Notably, both protein sets were also found in white matter, highlighting that the CNS may have similar immune shifts in response to the ketogenic diet.

Taken together, these findings indicate that a medical ketogenic diet leads to reprogramming rather than uniform activation or suppression of immune cell populations. Protein expression patterns and pathway analyses suggest a shift in T‐regs and NK cells from pro‐inflammatory or cytotoxic states toward more regulatory, tissue‐supportive phenotypes. Similarly, memory CD8^+^ T cells likely have reduced signatures of chronic activation alongside enhanced effector memory features, consistent with restored adaptive immune competence. Non‐Vδ2 γδ T cells also displayed bidirectional modulation, suggesting altered roles at the innate–adaptive interface and reduced IL‐17‐associated inflammatory signaling.

## 4 DISCUSSION

Our study provides the most comprehensive cross‐tissue characterization to date of the pro‐inflammatory immune phenotype associated with *APOE* ε4, integrating large‐scale clinical proteomic data with experimental validation in iPSC‐derived cortical organoids and a dietary intervention trial. We demonstrate that *APOE* ε4 carriers exhibit a conserved, allele dose‐dependent pro‐inflammatory immune signature across plasma, CSF, and brain tissue, independent of AD status. Importantly, this immune phenotype was also recapitulated in cortical organoids, emerging prior to the development of Aβ or tau pathology. This indicates that key features of the *APOE* ε4 immune signature arise early in disease pathogenesis and reflect an intrinsic, genotype‐driven mechanism of immune dysregulation. Cross‐tissue analyses further revealed a core set of systemic immune pathways involving broad innate and adaptive responses to infection, alongside distinct tissue‐specific signatures, including inflammatory signaling and BBB‐related interactions within the CNS. Notably, we show that this immune phenotype is not fixed. Short‐term treatment with a medical ketogenic diet in *APOE* ε4 carriers with AD partially reversed pro‐inflammatory signatures, promoting more regulatory and tissue‐supportive immune states. Together, these findings illuminate how peripheral and central tissue environments modulate *APOE* ε4‐driven immune responses, provide new insight into AD pathogenesis, and support the development of precision biomarkers and immunomodulatory interventions targeting early‐stage disease processes.

Our results are consistent with and extend prior work showing heightened peripheral immune responses in APOE ε4 carriers, including increased cytokine production after TLR2/4/5 activation,^3^ elevated TNF‐α, and IL‐6 after a lipopolysaccharide (LPS) challenge and increased susceptibility to inflammatory stressors such as sepsis^4^ and higher cytokine levels.^6^, ^42^, ^43^, ^44^ Peripheral blood mononuclear cell transcriptomic and epigenomic studies similarly report broad innate immune dysregulation, altered NF‐κB activation, increased chromatin accessibility in CD14^+^ monocytes, and clonally expanded CD8^+^ effector memory T cells.^45^ In line with this, our plasma signature is enriched for innate immune and antiviral responses, including TLR‐NF‐κB and interferon signaling, with allele dose‐dependent effects,^3^, ^45^ and we observe convergent viral response enrichment across plasma, CSF, and brain, supporting the emerging link between *APOE* ε4, viral reactivation, and AD risk.^46^ Across plasma and CSF, key proteins involved broadly in inflammatory processes included SPC25, LRRN1, S100A13, phosphoglycerate dehydrogenase (PHGDH), and FOXO1. SPC25 and LRRN1 have both been shown to modulate pro‐inflammatory processes in cancers.^47^, ^48^ Levels of S100A13 regulate cell surface‐associated IL‐1α and NF‐κB activity, playing a role in cellular senescence.^49^, ^50^ PHGDH regulates macrophage and microglial polarization^51^, ^52^ and plays a role in inflammatory responses of astrocytes.^53^ FOXO1 promotes IL‐1β^54^ and TLR4^55^ signaling in macrophages and regulates TGF‐β expression.^56^ In the CNS, our CSF and brain data suggest chronic inflammatory activation and BBB‐related immune pathway enrichment, consistent with *APOE* ε4 microglial dysfunction including impaired phagocytosis,^11^ disrupted autophagy with lipid droplet accumulation,^9^, ^10^ exaggerated inflammatory responses to Aβ,^10^, ^11^ BBB dysfunction,^57^, ^58^ and infiltration of peripheral immune cells such as IL‐17^+^ neutrophils.^7^ Pro‐inflammatory enrichment was stronger in the STG than the dlPFC, potentially reflecting greater vascularization and proximity to the CSF–blood interface. Mixed C‐reactive protein findings in *APOE* ε4 carriers,^42^, ^59^, ^60^, ^61^ including links to brain atrophy,^62^ suggest that single acute phase markers may not capture this chronic phenotype. Our conclusions are also limited by the lack of longitudinal data and cross‐platform proteomics, which constrained protein‐level comparisons and necessitated pathway‐level integration. However, concordant pathway signals across platforms provide orthogonal validation and support the utility of aptamer‐based profiling despite known proteoform sensitivity limits.^63^

A major strength of our study is the use of patient‐derived cortical organoids to model *APOE* ε4‐driven immune changes in human neural tissue before AD pathology emerges. While prior studies of *APOE* ε4 immunopathology largely focused on peripheral immune cells,^3^, ^4^, ^5^, ^6^, ^7^, ^42^, ^43^, ^44^, ^45^, ^62^, ^64^, ^65^ *post mortem* brain tissue,^8^, ^66^ or single iPSC‐derived cell types,^9^, ^10^, ^11^ our organoid model enables longitudinal study of *APOE* ε4 effects within a more complex network. We experimentally confirmed early activation of inflammatory pathways, including NF‐κB and DAM‐like signatures, prior to Aβ or tau pathology. This provides direct evidence that *APOE* ε4 immune dysregulation is intrinsic and genotype‐driven, rather than a secondary response to existing AD pathology. Importantly, our goal was not to isolate the singular effect of *APOE* ε4 in a genetically engineered system, but rather to establish the feasibility and translational relevance of modeling the *APOE* ε4 molecular phenotype in vitro and to determine its temporal emergence relative to hallmark AD pathology. As such, isogenic controls were not employed in the current study, as they would be less informative for assessing real‐world reproducibility of population‐derived proteomic signatures. Similarly, the use of multiple donor lines or diverse genetic backgrounds was not necessary, given that our organoid experiments were designed to validate robust molecular features identified across thousands of individuals in large clinical cohorts.^67^ This is a key distinction often overlooked in the field, where large‐scale cohort findings are frequently presented without experimental confirmation, an omission that undermines both mechanistic insight and translational potential.^68^, ^69^, ^70^, ^71^ Our findings address this gap directly, using a tractable human system to experimentally reproduce in vivo phenotypes. We propose, therefore, that experimental validation of high‐confidence cohort findings using patient‐derived models should become a standard step in the biomarker and drug development pipeline. Further, future studies should use the findings presented here as a starting point to determine mechanistic *APOE* ε4 immune pathways in AD, including through the use of isogenic controls. Although the control line was *APOE* ε3/ε2, cross‐cohort analyses show ε3/ε2 does not cluster separately from ε3/ε3 in plasma and is only partially separated in CSF. Overall, it remains far closer to ε3/ε3 than to ε3/ε4, supporting its use as a conservative comparator for *APOE* ε4 specific signatures. Future large‐scale organoid studies, however, would benefit from generating iPSCs from donors with differing *APOE* genotypes to study their respective molecular profiles. Furthermore, sourcing *APOE* ε4 iPSC lines from cognitively healthy donors remains inherently difficult, given the high lifetime risk of AD in this group. Nonetheless, this limitation is unlikely to confound our results, as we show that the *APOE* ε4 immune phenotype emerges prior to Aβ and tau accumulation, indicating that the presence or absence of clinical disease is irrelevant in this context.

The fact that *APOE* ε4 immune activation precedes Aβ and tau accumulation supports the view that these hallmark pathologies may be downstream consequences rather than primary disease drivers in *APOE* ε4 carriers. These insights challenge current therapeutic paradigms focused solely on Aβ and tau, targets that, despite effective clearance, have shown limited clinical benefit and, in some cases, worsening disease.^72^, ^73^, ^74^, ^75^ Instead, our findings suggest that tempering the persistent *APOE* ε4‐driven immune response through immunomodulation or anti‐inflammatory approaches will be critical for altering disease risk and progression in this high‐risk population. Importantly, this aligns with emerging evidence that vaccinations reduce AD risk,^76^, ^77^ including the shingles vaccines Zostavax^78^ and Shingrix,^79^ potentially by reducing reactivation of varicella zoster virus and/or inducing a virus‐specific immunomodulatory effect.^78^ Targeting immune dysregulation should therefore be prioritized in future precision medicine strategies for AD.

Our finding that a medical ketogenic diet partially reversed the *APOE* ε4 immune phenotype has important implications for therapeutic development. Twelve weeks of treatment led to the downregulation of chronic inflammatory signaling pathways, including NF‐κB, IL‐1, and TGF‐β, and upregulation of regulatory and phagocytic functions across T‐regs, NK cells, and memory CD8^+^ T cells. These changes suggest a shift away from maladaptive, chronic inflammation toward a more balanced and functional immune state. Importantly, these results demonstrate that the *APOE* ε4 immune phenotype is not a fixed, immutable trait but a plastic state that can be pharmacologically and metabolically reprogrammed. Showing that this genetically anchored immune signature is reversible in vivo provides critical proof of principle that *APOE* ε4‐associated immune dysregulation is a modifiable therapeutic target, rather than an unavoidable consequence of genotype. This supports the idea that immune modulation may be a viable disease‐modifying strategy, particularly if implemented early, before irreversible pathology has accumulated. Future studies would benefit from larger‐scale clinical trials of a medical ketogenic diet as well as other immunomodulatory therapies before the onset of AD in *APOE* ε4 carriers.

In conclusion, our findings establish that *APOE* ε4 drives a conserved pro‐inflammatory immune phenotype that emerges early, prior to the development of hallmark AD pathology. These results advance our understanding of *APOE* ε4‐mediated AD risk and challenge the prevailing Aβ‐ and tau‐centric therapeutic approaches, especially for *APOE* ε4 carriers. By demonstrating that immune dysregulation is an intrinsic, upstream feature of *APOE* ε4, and showing that it is modifiable, our study underscores the need to prioritize immunomodulatory and anti‐inflammatory strategies in precision medicine approaches for this high‐risk group. Notably, our human iPSC‐derived cortical organoid model recapitulated key immune features observed across clinical tissues, providing a tractable and translationally relevant system to experimentally validate cohort‐derived signatures. Moving forward, longitudinal studies and harmonized proteomic platforms will be critical to further delineate the temporal evolution of the *APOE* ε4 immune signature and to guide the development of targeted interventions aimed at altering disease trajectories in *APOE* ε4 carriers.

## CONFLICT OF INTEREST STATEMENT

The authors declare no competing interests or conflicts. Author disclosures are available in the Supporting Information.

## CONSENT STATEMENT

All participants gave written informed consent to participate.

## Supporting information




**Supporting Information**: alz71714‐supp‐0001‐ Tables.xlsx

**Supporting Information**: alz71714‐supp‐0002‐Figures.docx

**Supporting Information**: alz71714‐supp‐0003‐ICMJE.pdf
