# Supplementary material for: Cross‐tissue immune profiling of APOE ε4 reveals early dysregulation in Alzheimer's disease
Source: Alzheimers Dement. 2026 Aug 31;22(9):e71714. doi: 10.1002/alz.71714 (PMC13528450; doi:10.1002/alz.71714)
Supplement: Supplementary file 2 [file ALZ-22-e71714-s003.docx]

**Supplementary Fig. 1. Principal component analyses of plasma and cerebrospinal fluid proteins.** Plasma proteins principal component analysis (PCA) of (**a**) all 6,340 proteins, (**b**) nine identified *APOE* ε4 proteins showing clustering based on presence (+) or absence (-) of an *APOE* ε4 allele, and (**c**) nine identified *APOE* ε4 proteins showing clustering based on the number of *APOE* ε4 alleles. Cerebrospinal fluid (CSF) PCA of (**d**) all 6,340 proteins, (**e**) 51 identified *APOE* ε4 proteins showing clustering based on presence (+) or absence (-) of an *APOE* ε4 allele, and (**e**) 51 identified *APOE* ε4 proteins showing clustering based on the number of *APOE* ε4 alleles. Abbreviations: AD: Alzheimer’s disease; NI: non-impaired control.

**Supplementary Fig. 2. Functional enrichment analysis of up- and down-regulated cerebrospinal fluid (CSF) *APOE* ε4 proteins.** (**a**) Significantly (FDR < 0.05) enriched KEGG immune pathways in CSF *APOE* ε4 proteins. (**b**) Significantly (FDR < 0.05) enriched Reactome immune pathways in CSF *APOE* ε4 proteins. Red bars represent enriched pathways for up-regulated proteins. Blue bars represent enriched pathways for down-regulated proteins.

**Supplementary Fig. 3. Functional enrichment analysis of up- and down-regulated dorsolateral prefrontal cortex (dlPFC) and superior temporal gyrus (STG) *APOE* ε4 proteins.** (**a**-**b**) Significantly (FDR < 0.05) enriched (**a**) KEGG and (**b**) Reactome immune pathways in dlPSC *APOE* ε4 proteins. (**c**-**d**) Significantly (FDR < 0.05) enriched (**c**) KEGG and (**d**) Reactome immune pathways in STG *APOE* ε4 proteins. Red bars represent enriched pathways for up-regulated proteins. Blue bars represent enriched pathways for down-regulated proteins.

**Supplementary Fig. 4.** **Representative immunofluorescent image of a cortical organoid.** Images show staining for DAPI (nuclei), microtubule associated protein 2 (MAP2; neurons), glial fibrillary acidic protein (GFAP; astrocytes), ionized calcium-binding adapter molecule 1 (IBA1; microglia), and an overlay of all four markers. Scale bar represents 10μm.

**Supplementary Fig. 5. Functional enrichment analysis of up- and down-regulated stem cell-derived cortical organoid *APOE* ε4 proteins at four and eight weeks.** (**a**-**b**) Significantly (FDR < 0.05) enriched (**a**) KEGG and (**b**) Reactome immune pathways in stem cell-derived cortical organoid *APOE* ε4 proteins at four weeks. (**c**-**d**) Significantly (FDR < 0.05) enriched (**c**) KEGG and (**d**) Reactome immune pathways in stem cell-derived cortical organoid *APOE* ε4 proteins at eight weeks. Red bars represent enriched pathways for up-regulated proteins. Blue bars represent enriched pathways for down-regulated proteins.
